# A Novel High Performance Liquid Chromatographic Method for Determination of Nystatin in Pharmaceutical Formulations by Box–Behnken Statistical Experiment Design

**Published:** 2015

**Authors:** Farnaz Shokraneh, Ramin Asgharian, Assem Abdollahpour, Mehdi Ramin, Ali Montaseri, Arash Mahboubi

**Affiliations:** a *Department of Pharmaceutics, Pharmaceutical Sciences Branch, Islamic Azad University (IAU), Tehran, Iran.*; b *Quality Assurance Lab, Jaber Ebne Hayyan Pharmaceutical Company, Tehran, Iran.*; c *Afa Chemi Pharmaceutical Company, Tehran, Iran.*; d *Department of Pharmaceutics, School of Pharmacy, Shahid Beheshti University of Medical Sciences, Tehran, Iran.*

**Keywords:** Nystatin, High performance liquid chromatography, Box–Behnken experimental design

## Abstract

In this study a novel High Performance Liquid Chromatography for the assay of nystatin in oral and vaginal tablets were optimized and validated using Box–Behnken experimental design. The method was performed in the isocratic mode on a RP-18 column (30 °C) using a mobile phase consisting of ammonium acetate 0.05 M buffer/ Methanol mixture (30:70) and a flow-rate of 1.0 mL/min. The specificity, linearity, precision, accuracy, LOD and LOQ of the method were validated. The method was linear over the range of 5–500 µg/mL with an acceptable correlation coefficient (r^2^ = 0.9996). The method’s limit of detection (LOD) and quantification (LOQ) were 0.01 and 0.025 µg/mL respectively. The results indicate that this validated method can be used as an alternative method for assay of nystatin.

## Introduction

Nystatin as a polyene antifungal medication is naturally derived from *Streptomyces noursei*. Many molds and yeast are sensitive to nystatin and it has been used for treatment of infections caused by *Candida*, *Cryptococcus*, *Histoplasma*, *Blastomyces *and *Aspergillus spp* (1, 2). It is now widely prescribed for fungal infections (3). Nystatin could be safely adminstrated orally as well as topically (4).

According to current Pharmacopeia, including European and United States Pharmacopeia, nystatin as well as many other antibiotics is assayed by bioassay method based on agar diffusion (5, 6).

 The existence of several systems to express the potency of antibiotics leads to confusion and more over the use of potency to express the content of bulk products can lead to difficulties in the interpretation of the content of pharmaceutical preparations. Several HPLC and Spectrophotometric methods have been proposed and investigated to measure nystatin in biological samples (*e.g*. plasma and saliva); however the extraction for various concentrations is time consuming, tedious with limited detection with UV monitoring and the LC-MS method (7-11).

In this study a simple and robust HPLC procedure for the measurement of nystatin in bulk, oral and vaginal tablets is developed and validated. The HPLC method was developed and optimized using Box-Behnken design in order to obtain the amounts of the parameters and also to investigate the interaction between the parameters.

## Experimental

*Reagents and chemicals*

Methanol, Ammonium Acetate and Acetic Acid were purchased from Merck (Germany). Solutions were prepared by deionized water, produced by a MilliQ water purification system from Milipore (Bedford, MA, US). Nystatin was purchased from CIPLA (Bombay, India). Amphotericin B an internal standard was purchased from Sigma–Aldrich, Germany.

*Instrument*

The analyses were performed on HPLC instrument consisted of a computer-controlled system with ChromGate software and Knauer solvent organizer K-1500, Knauer low pressure HPLC pump K-1001 and PDA detector K-2800 operated at 304 nm for analysis. Chromatographic separations were run on a Teknokroma 250 mm × 4.6 mm Symmetry 5 μm, C18 analytical column. The chromatographic experiments were performed under isocratic elution. Methanol and 0.05 M ammonium acetate buffer with in proportion of 70:30 v/v were used as the mobile phase consisted of. The mixture pH was adjusted to 5.0 with glacial acetic acid. The mobile phase was degassed by sonication under low vacuum before use. The injection volume was 20 µL using a RHEODYNE (Rohnert Park, CA, USA) model 7725i manual injector with the flow rate of 1.0 mL/min. The retention time of nystatin was approximately 16 min. Samples and spiked standards were analyzed after preparation. Concentrations were calculated from calibration curves constructed by plotting peak heights (AUC) against concentrations of standards containing graded amount of nystatin.

*Stock and working standard solutions*

Nystatin stock solution (600 µg/mL) was prepared in mobile phase. This solution was then diluted with the mobile phase in order to obtain the working standard solutions at concentrations of 5, 10, 25, 50, 100, 200, 300, 400 and 500 µg/mL. Twenty five µg/mL of amphotericin B (as an internal standard) was added into all of the working standard solutions and the mixture was gently shaken.

*Preparation of sample solutions*

For the preparation of sample solution, 10 tablets were taken and weighed individually. Average weight was calculated and finely powdered. Appropriate portion of this fine powder equivalent to 5 mg of nystatin was weighed and transferred to a 25 mL volumetric flask. This was dissolved and made up to the 25 mL with mobile phase. The solution was sonicated for 10 minutes before filtration by a 0.45 μm membrane filter. 

## Results and Discussion

*Method development and optimization*

For initial study, various types of mobile phases (solvents) (acetonitrile, ethanol, water with ammonium acetate buffer) were studied to optimize the method. The flow rate and column oven temperature were selected with regards to the back pressure and analysis time as well. When studied was performed with acetonitrile and ethanol, poor resolution and unsuitable peak shape between nystatin and amphotericin B and higher peak tailing were observed.

In order to optimization of the method and also to study the possible interaction between the parameters, Box-Behnken design was used (12). Levels and the parameters were based on results from the initial study. A Box-Behnken statistical design with 5 factors, 3 levels and 46 runs was selected for the optimization study and the observed responses are given in Table 1. The experimental design consists of a set of points lying at the midpoint of each edge and the replicated center point of multidimensional cube. pH (A), Concentration of ammonium acetate buffer (B), flow rate (C), ratio of mobile phase (D) and column oven temperature (E) were selected as independent variables in Box-Behnken design. Resolution was taken as response for further analysis. Based on the experimental design, the combinations of factors yielded different mean responses. Table 1 summarizes the experimental runs, the levels of experimental units and their factor combinations in the study as well as response. Using Box-Behnken design, the model was fitted to the data. Regression analysis of the data was carried out in Stat Graphics Plus 5.1 by a special cubic model.

**Table 1:** Variables in Box-Behnken design and results for each experimental runs

**Factor Key**							**levels**		
							** -1 0 +1 **		
pH Buffer concentration Flow Rate Ratio of mobile phase Column Temperature				A			4	5	6
				B			0.01	0.05	0.1
				C			0.8	1	1.2
				D			60	70	80
				E			25	30	35
**Run**	**A**	**B**	**C**		**D**	**E**	**Response ** **(Relative Resolution)**		
1	0	1	0		0	1	17.22		
2	-1	-1	0		0	0	18.02		
3	1	1	0		0	0	13.71		
4	0	0	-1		-1	0	0		
5	0	0	-1		0	1	17.40		
6	0	1	0		0	-1	19.17		
7	0	-1	1		0	0	16.99		
8	0	1	-1		0	0	18.94		
9	0	0	-1		0	-1	19.93		
10	0	1	0		-1	0	0		
11	0	0	0		0	0	8.25		
12	0	0	-1		1	0	19.04		
13	0	-1	0		-1	0	0		
14	0	0	0		0	0	18.34		
15	1	0	0		-1	0	0		
16	-1	0	-1		0	0	6.11		
17	0	0	0		-1	1	18.87		
18	0	0	1		0	-1	17.81		
19	0	1	1		0	0	17.78		
20	0	0	1		-1	0	0		
21	0	0	0		-1	-1	0		
22	0	-1	0		0	-1	19.10		
23	1	0	0		0	1	15.09		
24	0	-1	0		0	1	17.47		
25	1	0	1		0	0	15.78		
26	0	0	1		0	1	16.68		
27	0	0	0		1	1	7.54		
28	-1	0	0		1	0	5.66		
29	-1	0	0		0	1	14.54		
30	1	-1	0		0	0	18.46		
31	0	0	0		-1	1	0		
32	0	0	0		0	0	18.34		
33	0	-1	0		1	0	8.69		
34	0	-1	-1		0	0	18.95		
35	1	0	0		0	-1	17.19		
36	-1	0	1		0	0	14.66		
37	0	0	0		0	0	18.34		
38	1	0	0		1	0	8.50		
39	0	1	0		1	0	7.43		
40	0	0	0		0	0	18.34		
41	0	0	1		1	0	8.03		
42	-1	0	0		-1	0	0		
43	1	0	-1		0	0	15.94		
44	0	0	0		0	0	18.34		
45	-1	0	0		0	-1	15.05		
46	-1	1	0		0	0	13.75		

**Figure 1:**
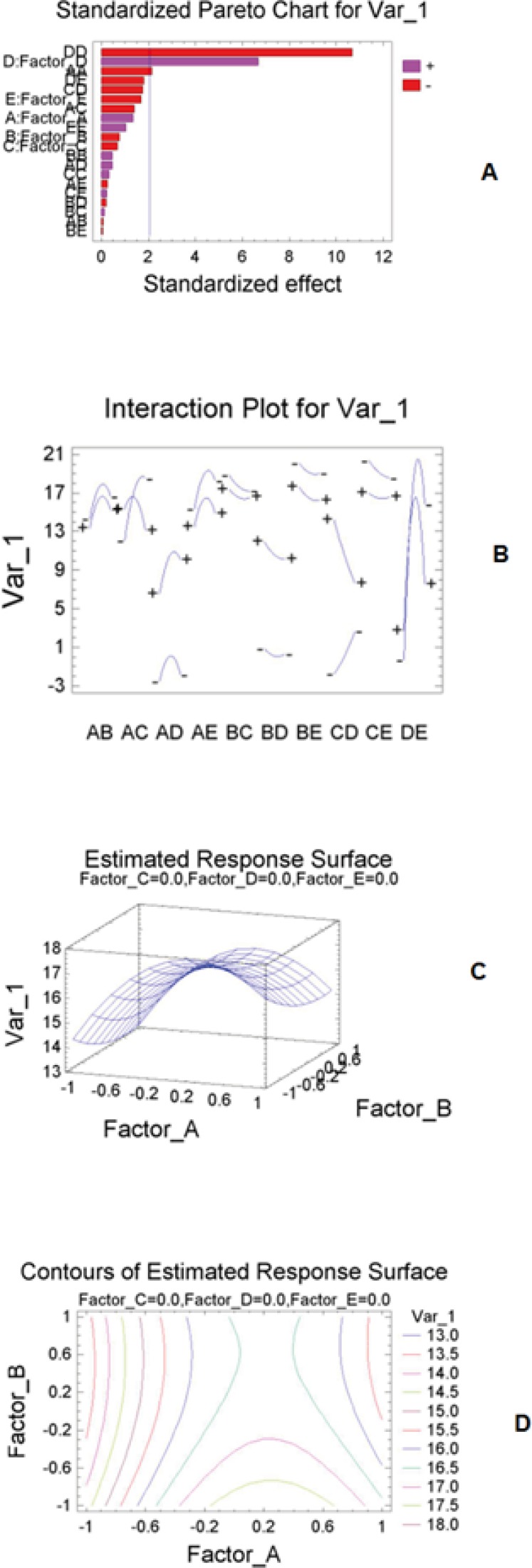
(A) Pareto chart of the main effects for chromatography method; (B) interaction plot; (C) estimated response surface; and (D) contours plot obtained

The Standard Pareto chart was used to assess the impact of each parameter in response to the chromatographic method. The normalized results of the experimental design, evaluated at a 5% of significance, were analyzed by a standardized Pareto chart, which showing a frequency histogram where, the length of each bar on the chart is proportional to the absolute value of its associated estimated effect or the standardized effect (Figure 1A). The standardized effect is the estimated effect divided by its standard error, which is equivalent to computing a t-statistic for each effect. The plot vertical line judges the effects that are statistically significant. Based on the Box-Behnken design results, mobile phase ratio has maximum effect on the optimization methods (Figure 1A). The interaction plot mentioned that there is an interaction between pH and flow rate (Figure 1B). As one can see from the estimated responses surface (Figure 1C). The optimum conditions were in the position of maximum levels of the surface and the lines of the estimated responses surface confirmed the model and optimum conditions (Figure 1D). The optimum conditions obtained by Box-Behnken design are as follows: The mobile phase, methanol -0.05 M ammonium acetate buffer (70:30 v/v); pH 5.0; temperature of 30 °C; flow rate, 1.0 mL min^-1^.

*Method validation*

According to the ICH-Guidelines on the validation of analytical methods Q2A and Q2B the method validation demonstrated the specificity, linearity, limit of detection, limit of quantification, precision, and accuracy (13, 14).

*Specificity*

**Figure 2:**
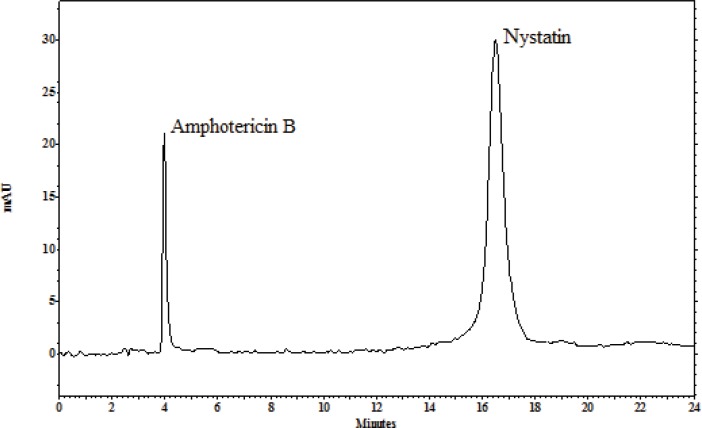
Typical chromatograms for separation conditions: amphotericin B at 4 min and nystatin at 16 min.

The ability of these methods to set apart the peaks indicates the methods specificity. UV detector and retention times of the internal standard and nystatin were used to recognize the peaks in the chromatograms with the current separation conditions: amphotericin B at 5 min, nystatin at 15 min (Figure 2). 

*Linearity*

Injection of standard solution in nine concentration levels over a wide concentration range (5, 10, 25, 50, 100, 200, 300, 400, 500 µg/mL) was utilized to recognize the linearity of the method. The coefficients of correlation (r*>*0.999) exhibited the significant relationship between peak area and the concentration of nystatin. (Table 2) gives information on the calibration range, regression equation, the coefficient of correlation, and the LOD and LOQ of nystatin. 

**Table 2:** Overview of the linearity data for assay of nystatin

**Analyte (nystatin)**	
Calibration range [µg/mL]	5-500 [µg/mL]
y-intercept	146641 44889
Slope	
Coefficient of correlation	0.9996
LOD [µg/mL]	0.01
LOQ [µg/mL]	0.025

*Precision of the chromatographic method*

Two different ways including repeatability as an intra assay and intermediate precision as inter assay were used to confirm the precision of the HPLC. The repeatability was settled by analyzing six repeated injections of the standard solution. For the intermediate precision, six tests were repeated on a different day by a different analyst. The relative standard deviation values (RSD) of the repeatability and intermediate precision must be less than 1% and demonstrated that the HPLC method is precise (Table 3). 

**Table 3:** Precision and accuracy by recovery of the chromatographic method

**Concentration level (%)**	**RSD%**	
	**Intra-assay ** **(1 day)** **(n=6)**	**Inter-assay (3 days)** **(n=18)**
50%	0.85	0.10
100%	0.68	0.54
150%	0.92	0.88
Average	0.82	0.51

*Accuracy*

The accuracy of the procedure was assessed by comparing the analyte amount determined versus the known amount spiked at three different concentration levels (50%, 100%, and 150%) with 3 replicates (n=3) for each concentration level investigated. Each sample was injected three times and analyzed according to the method that was previously described (Table 3). These results confirm that the method is accurate for nystatin with recovery rates of 92-100%. 

The proposed chromatographic method was applied to assay of nystatin in pharmaceutical samples are summarized in Table 4. 

**Table 4:** Results obtained for assay of nystatin in real samples and comparison with standard method.

**Real samples**	**Assay results**	
	**HPLC assay** **(IU/Tablet)**	**Microbial assay** **(IU/Tablet)**
Vaginal Tablet 100,000 IU	114328	101250
Oral Tablet 500,000 IU	499012	520000

## Conclusions

In this study we have demonstrated a sensitive and selective HPLC method for the determination of nystatin in pharmaceutical formulations. The proposed method is specific and there is no interference from any of the sample components. The method is robust, accurate, precise and linear over a wide range of concentrations and is suitable for routine quality control.

## References

[1] Arikan S, Ostrosky-Zeichner L, Lozano-Chiu M, Paetznick V, Gordon D, Wallace T, Rex JH (2002). In-vitro activity of nystatin compared with those of liposomal nystatin, amphotericin B, and fluconazole against clinical Candida isolates. J. Clin. Microbiol.

[2] Offner F, Krcmery V, Boogaerts M, Doyen C, Engelhard D, Ribaud P, Cordonnier C, De Pauw B, Durrant S, Marie JP, Moreau P, Guiot H, Samonis G, Sylvester R, Herbrecht R (2004). Liposomal nystatin in patients with invasive aspergillosis refractory to or intolerant of amphotericin B. Antimicrobial. Agents Chemother.

[3] Ostrosky-Zeichner L, Bazemore S, Paetznick VL, Rodriguez JR, Chen E, Wallace T, Cossum P, Rex JH (2001). Differential antifungal activity of isomeric forms of nystatin. Antimicrobial. Agents Chemother.

[4] Razonable RR, Henault M, Watson HL, Paya CV (2005). Nystatin induces secretion of interleukin (IL)-1, IL-8, and tumor necrosis factor alpha by a toll-like receptor-dependent mechanism. Antimicrobial. Agents Chemother.

[5] (2009). British Pharmacopoeia.

[6] Gharia T, Kobarfard F, Mortazavia SA (2013). Development of a simple RP-HPLC-UV method for determination of azithromycin in bulk and pharmaceutical dosage forms as an alternative to the USP method. Iran. J. Pharm. Res.

[7] Groll AH, Mickiene D, Werner K, Piscitelli SC, Walsh TJ (1999). High-performance liquid chromatographic determination of liposomal nystatin in plasma and tissues for pharmacokinetic and tissue distribution studies. J. Chromatogr. B Biomed. Sci. Appl.

[8] Cione APP, Liberale MJ, Da Silva PM (2010). Development and validation of an HPLC method for stability evaluation of nystatin. Brazilian J. Pharm. Sci.

[9] Scheuch E, Giessmann T, Siegmund W (2006). Quantitative determination of nystatin in human plasma using LC-MS after inhalative administration in healthy subjects. J. Chromatogr. B Analyt. Technol. Biomed. Life Sci.

[10] Llabot JM, Allemandi DA, Manzo RH, Longhi MR (2007). HPLC method for the determination of nystatin in saliva for application in clinical studies. J. Pharm. Biomed. Anal.

[11] Lemus Gallego JM, Perez AJ (2002). Spectrophotometric determination of hydrocortisone, nystatin and oxytetracycline in synthetic and pharmaceutical preparations based on various univariate and multivariate methods. Anal. Chim. Acta.

[12] GEP Box, DW Behnken (1960). Some new three level designs for the study of quantitative variables. Technometrics.

[13] (1996). ICH, Q2B, Hamonised Tripartite Guideline, Validation of Analytical Procedure: Methodology, IFPMA, in: Proceedings of the International Conference on Harmonization.

[14] (1994). ICH, Q2A, Hamonised Tripartite Guideline, Test on Validation of Analytical Procedures, IFPMA, in: Proceedings of the International Conference on Harmonization.

